# Human-machine Interface using functional electrostimulation and inertial sensors for lower limb rehabilitation in spinal cord injury individuals: a proof of concept

**DOI:** 10.1007/s11517-025-03501-z

**Published:** 2026-01-24

**Authors:** Luiz Henrique Bertucci Borges, Cristian Felipe Blanco-Díaz, Bruno Henrique e Silva Bezerra, Caroline Cunha do Espírito Santo, Teodiano Bastos-Filho, Denis Delisle-Rodriguez, André Felipe Oliveira de Azevedo Dantas

**Affiliations:** 1 Postgraduate Program in Neuroengineering, Edmond and Lily Safra International Institute of Neuroscience, Santos Dumont Institute, Av. Alberto Santos Dumont, 1560 – Zona Rural, Macaiba, Rio Grande do Norte 59280-000 Brazil; 2 Postgraduate Program in Electrical Engineering, Federal University of Espírito Santo (UFES), Vitória, Espírito Santo Brazil

**Keywords:** Electroencephalography, Human-machine interface, Functional electrical stimulation, Inertial motion unit, Lower-limb rehabilitation, Spinal cord injury

## Abstract

**Abstract:**

A spinal cord injury (SCI) is a neurological disorder that impairs motor and physiological functions and leads to a reduced quality of life and autonomy for the person affected. In this scenario, human-machine interfaces (HMIs) have emerged as an effective tool to leverage residual motor capabilities and benefit injured persons. This work aims to develop a closed-loop HMI system for lower-limb rehabilitation composed of an in-house multi-channel Functional Electrical Stimulation (FES), which is activated by considering gait and pedaling cycles measured by an Inertial Measurement Unit. Two experiments were conducted with individuals suffering partial SCI who performed cycling and walking activities by using our proposed HMI, while inertial and electroencephalography signals were collected for further analysis and validation. Relative power changes were observed in mu (8–13 Hz) and high beta (20–30 Hz) bands over the foot area (Cz location), comparing both FES and non-FES conditions during gait and pedaling. This comparison also showed that the volunteers performed physical activities with greater speed and cadence by using the proposed HMI system, which correctly identified the movement phases.

**Graphical abstract:**

A spinal cord injury (SCI) is a neurological disorder that impairs motor and physiological functions, leading to reduced quality of life and autonomy for affected individuals. In this context, human–machine interfaces (HMIs) have emerged as effective tools to enhance residual motor capabilities and support rehabilitation.

This study aims to develop a closed-loop HMI system for lower-limb rehabilitation composed of an in-house multi-channel Functional Electrical Stimulation (FES) device, activated based on gait and pedaling cycles measured by an Inertial Measurement Unit (IMU). Two experiments were conducted with individuals with partial SCI who performed cycling and walking tasks using the proposed HMI system, while inertial and electroencephalography (EEG) signals were recorded for further analysis and validation.

Relative power changes were observed in the mu (8–13 Hz) and high beta (20–30 Hz) bands over the foot area (Cz location) when comparing FES and non-FES conditions during gait and pedaling. This comparison also revealed that participants performed physical activities with greater speed and cadence when using the proposed HMI system, which successfully identified movement phases in real time.

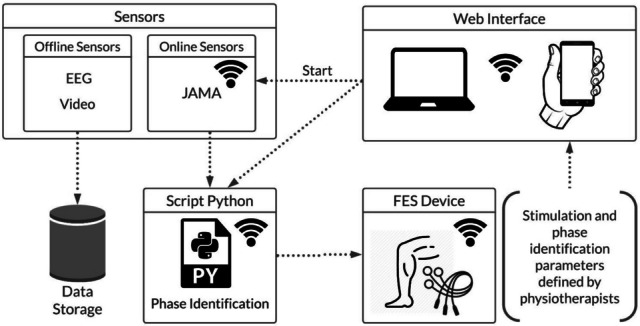

## Introduction

Incomplete or complete spinal cord injury (SCI) is a neurological event that impairs motor and physiological function and affects 500,000 people worldwide each year. The physical and physiological limitations that occur after SCI significantly degrade the quality of life and autonomy of individuals [2]. In this context, residual axonal connections have been documented in individuals post-injury, facilitating functional restoration by preserving communication pathways within affected regions [17]. Hence, novel and advanced neurorehabilitation methods may induce the sensorimotor recovery and neuroplasticity [14]. Indeed, the execution of active movements during physical training plays a crucial role in promoting the reorganization of neural pathways, thereby improving motor skills [10, 47].

Therapies that promote patient participation and interaction have demonstrated improving in the sensorimotor rehabilitation of people with neural impairments [14, 24]. For instance, human-machine interfaces (HMIs) have emerged as an effective tool for utilizing the remaining motor skills of people with severe disabilities and converting them into the control system of rehabilitation or assistance devices. Inertial Measurement Units, surface Electromyography (sEMG), and electroencephalography (EEG) are widely used to decode patients’ intentions into end-effector drivers in an almost natural way, such as exoskeletons, functional electrical stimulation (FES), and passive devices, allowing them to interact with the environment [1, 35, 39]. Invasive HMIs employing implanted electrodes provide high-quality cortical signal acquisition; however, they entail higher costs and require surgical implantation procedures. Noninvasive HMIs, in contrast, use surface EEG, which provides lower signal precision but offers reduced cost and ease of implementation [11, 48].

Some studies have suggested lower-limb rehabilitation by employing robotic devices for passive movement, such as robotic orthosis attached to a chair [49], or a motorized pedal approach to generate cyclic movements [37]. However, FES has emerged as a technique involving residual neural path and artificial current to activate residual neuromuscular structures, generating movements. For instance, in [45], the authors proposed a rehabilitation system in which pedaling movements were controlled with FES in a closed loop. In [31], the authors claimed that gait protocols with FES could increase muscle strength and improved walking performance. Consequently, different HMI approaches based on FES have been proposed in rehabilitation or assistive contexts. For instance, several reviews has showed FES applications in post-stroke patients triggered wit EEG or inertial sensors for limbs rehabilitation or assistance [23, 39, 45]. However, experimental setup applied with SCI patients are scarce and there is a predominance of approaches to upper limbs, rather than lower limbs.

Therefore, the search for alternative strategies that allow an effective rehabilitation of lower limbs for the performance of daily tasks remains a challenge. For instance, Ren et al. proposed a soccer virtual reality, employing FES in a leg to improve motor imagery (MI) classification [36]. Le Guillou et al. realized a pedaling phase recognition system, which can be used to synchronously deliver FES to the legs [25]. CoelhoMagalahes et al. designed a closed-loop control strategy to adjust FES amplitudes during pedaling [9]. Sijobert et al. employed a multichannel FES and sensors based on plantar pressure and IMUs to activate FES in one leg during gait in post-stroke patients [41]. Li et al. Implemented a neural network-based controller and sEMG to actuate foot dorsiflexion that was used by people with foot drop [26].

In this context, lower-limb movements require the integrated involvement and coordination of different muscle groups, which is a challenge for FES-based HMIs. A well-controlled and synchronized multichannel FES approach would allow more natural actions. For instance, Muller et al. [30] proposed a neuroprosthesis for automatic gait control by using IMU sensors, which faced difficulties in recognizing the gait phases. Despite the significant progress in FES-based HMI systems for lower-limb rehabilitation of people with severe neuromotor disabilities, there are still some scientific problems and challenges.

For instance, one of the main issues is the limited integration of FES devices with other systems, since many existing approaches enable only non-synchronized or manually triggered activation of stimulation channels, rather than a control synchronized and integrated with external systems. [28]. This restricts the possibility of achieving more natural, synchronized, and functional movements during complex tasks such as walking and pedaling. Nevertheless, the quality of EEG signals, especially during walking, can be affected by movement artifacts and the poor signal-to-noise ratio (SNR), as well as non-physiological and physiological interference [6, 44]. In addition, lower limbs detection by EEG may be limited by the leg area of the motor cortex which is deeply located in the brain (around 1–4 cm from the surface). Therefore, EEG could not accurately record this activity, and its signals can be highly influenced by activity occurring near the surface of the skull [16].

This work is a further development of our previous works [13], which aim to develop a closed-loop system with multi-channel HMI FES-based devices for lower-limb rehabilitation by using IMU for automatic detection of movement phases. We hypothesize that the proposed HMI will provide more natural movements during walking or cycling by accurately detecting each movement phase and performing electrical stimulation with appropriate parameters. Furthermore, relative EEG power changes during pedaling and walking for mu and beta rhythms in the motor cortex were observed, where our findings suggested a promising tool for the implementation of FES-based HMIs for neurorehabilitation. The main contributions of this work are:

1. We have designed a non-invasive FES-based HMI that allows coordinated activation of different channels to assist movement of people with neurological disorders. Unlike systems reported in the literature and in previous studies [9, 25, 26, 30, 41], we use a multichannel strategy and IMU for a complete assistance in both legs during four phases of gait or pedaling, which would allow a closer-to-natural motion and a more personalized rehabilitation.
2. We conducted a proof-of-concept trial with two post-SCI patients undergoing pedaling and gait-assist interventions, both of whom demonstrated improved movement characteristics using our HMI.
3. EEG signals were acquired and analyzed, where it was possible to observe the influence of FES specifically in the beta band (13–35 Hz), whose results are similar to those in the literature with healthy subjects in cycling and walking [5, 44].

## Materials

The BrainVision® V-amp 16 is used here to record cortical activity with sampling rate at 512 Hz, using 16 EEG locations (Fp1, Fp2, F3, F4, FC1, FC2, C3, Cz, C4, CP1, CP2, P3, P4, P7, P8 and Pz) according to the international 10–20 system, covering the frontal region, and the primary and supplementary motor cortex [14, 20, 37]. The reference and ground electrodes were positioned at locations Fz and Fpz, respectively. The electrode’s impedance was maintained below 10 kΩ.

ZeroG was used, a dynamic body weight support system that compensates for weaknesses and coordination deficiencies and enables earlier intensive therapy sessions [22]. This allows the percentage of suspended weight to be controlled, with the value adjustable according to the subject’s condition and rehabilitation progress. The system provides protection against falls and analyzes speed variables. The inertial sensor used for phase identification is a compact version of a Joint Angle Measurement and Acquisition (JAMA) device [3]. In addition to acquiring data for posterior analysis, the sensor is responsible for identifying phases by converting acceleration data into *θ* angles.

**Fig. 1:**
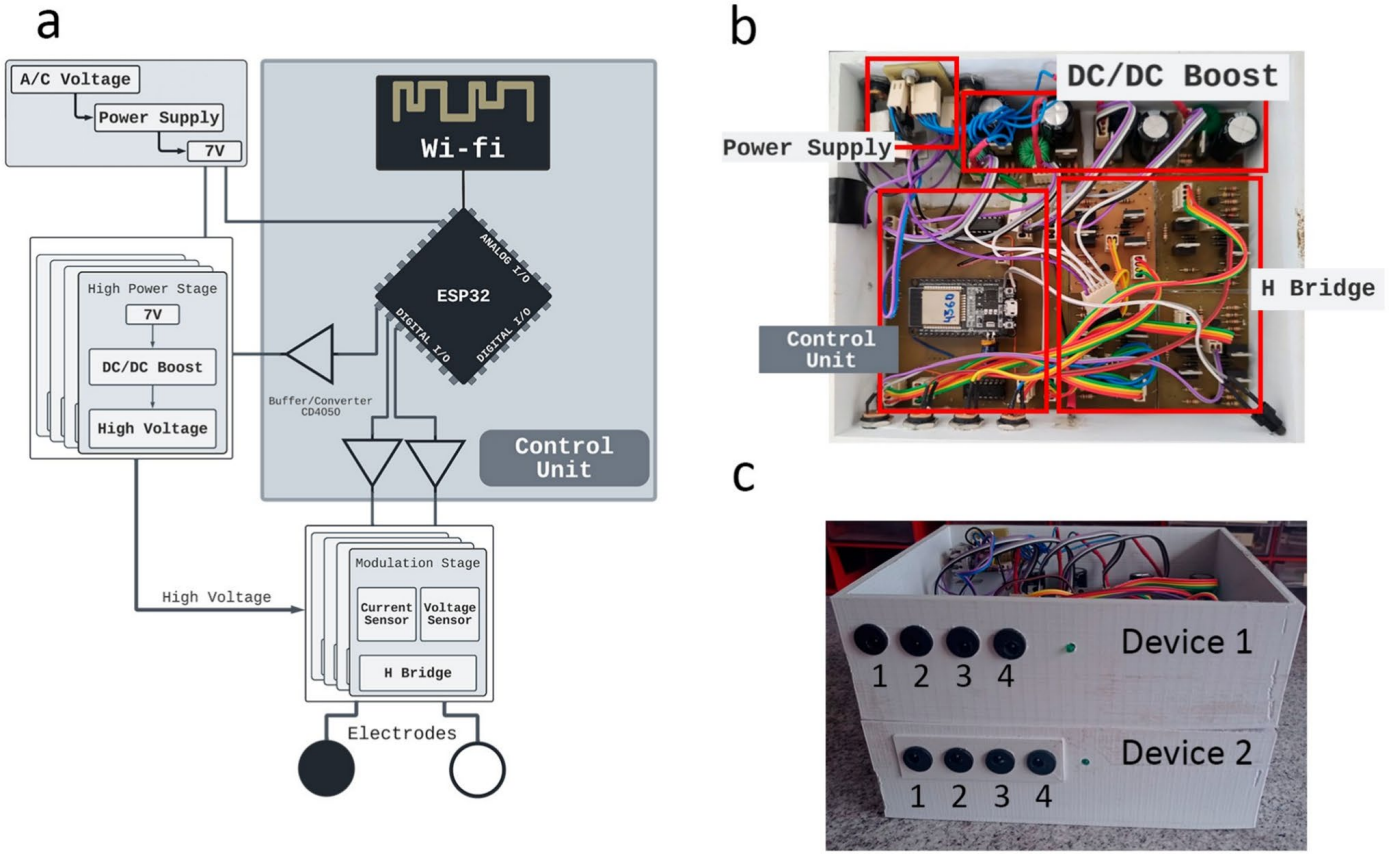
**a** Hardware by [13]; **b** Hardware description; **c** Two 4-channel IoT FES devices

The FES device used in this research represents an advance in hardware, as previously presented in [13]. The multi-channel FES circuit was designed with an upconverter, H-bridge, and processing module, as shown in Fig. 1(a). The processing module is a microcontroller (ESP32-DevKitC) that manages the stimulation signals by using pulse-width modulation (PWM) (to control the voltage on the booster and modulate the signal that controls the H-bridge). A processing module can control up to four stimulation channels (four boost converters and four H-bridges) to fulfill the multi-channel proposal. In the version developed in this work, a set of two FES devices, as shown in Fig. 1(b) has been proposed, including a total of eight stimulation channels controlled independently via Message Queuing Telemetry Transport (MQTT).

## Methods

### Human-machine interface

**Fig. 2:**
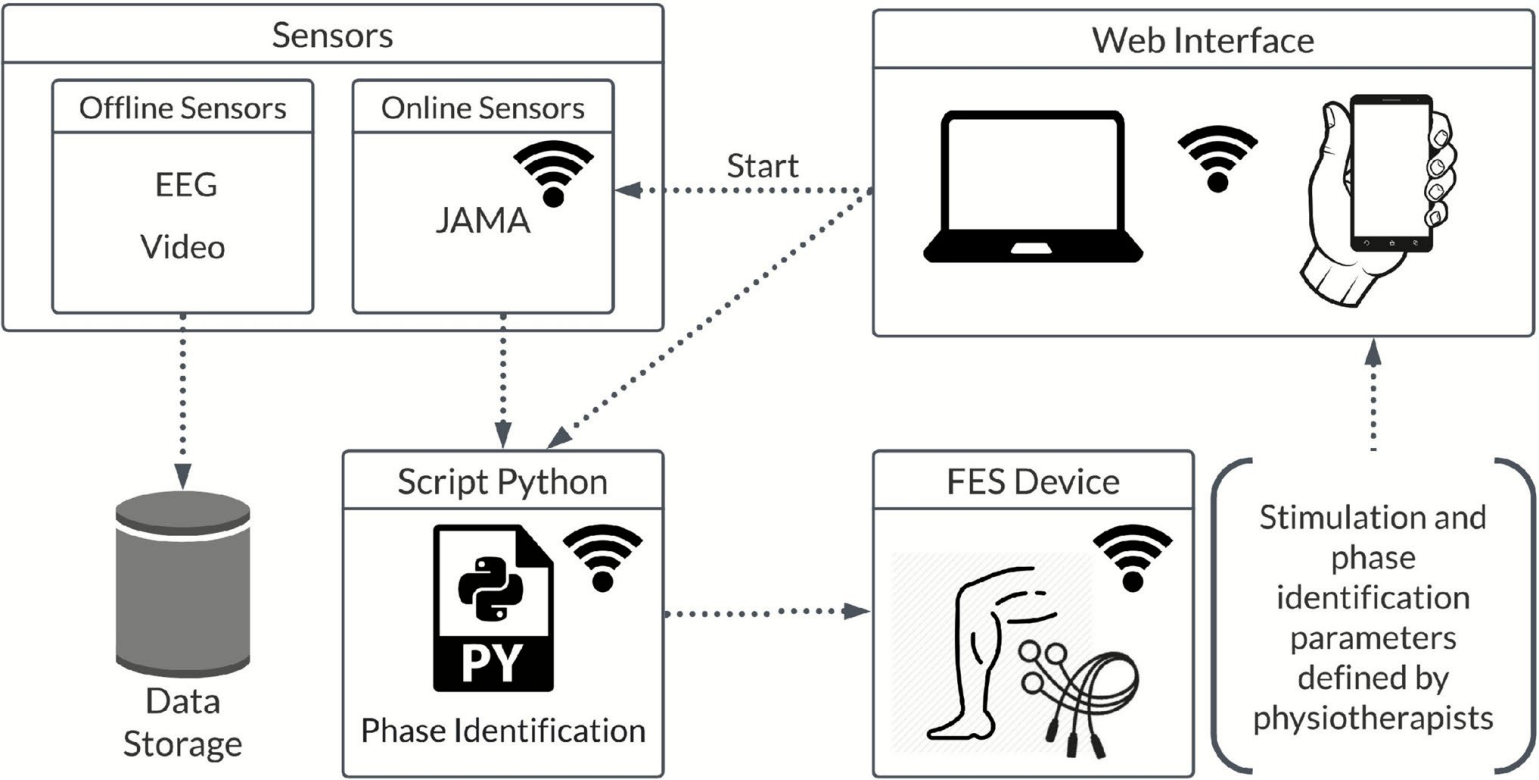
System Overview

The HMI system used in the proof of concept consists of two low-cost 4-channel FES devices and an IMU sensor, which were used for electrical stimulation and motion intention detection, respectively, operating in a closed-loop. The data were divided into online and offline groups, as shown in Fig. 2. The offline data are stored for further analysis, whereas the online data are stored and transferred in real-time between the system’s devices to be used in a closed-loop control. A Python script manages the proposed system, and a local web application using an MQTT web socket. This technique allows the therapist to control the stimulation parameters (for each channel individually), the IMU angle ranges, and emergency stops in real-time if needed. The MQTT network was implemented for security and low latency in data transmission between the devices. The therapist starts the protocol via the WEB browser (notebook or smartphone) by sending a “start” command to the JAMA device. This sent the inertial sensor data to the Python script. In the same script, the acceleration data from the inertial sensor are converted into an angle *θ* that is used to identify the phases of walking or cycling. The control action is then sent to the FES device to activate the stimulation channels for each phase.

The HMI is designed to control the subject’s residual motor activity based on the motion signal detected by the IMU sensor. For this purpose, the 2-axis accelerometer data (*a*_*x*_ and *a*_*y*_) are converted into an angle *θ*, considering *θ* = *arctan*(*a*_*x*_*/a*_*y*_). The angular range of *θ* can be divided into two or four phases depending on the motor task (pedaling or walking) and linked to each movement phase. Figure 2 shows that the therapist specifies the muscle groups to be activated and the intensity and frequency of stimulation in the web application, considering the protocol, participant’s condition, and prior assessment by the medical team.

### Patient’s safety

Hardware and software are used to ensure patient safety. The first provides an emergency button placed directly on the patient’s hand so that they can switch off the FES device if they feel uncomfortable. In short, pressing the emergency button immediately cuts power to the devices and quickly terminates the stimulation in progress. As a second alternative via hardware, the boost converter discharge system can be activated by switching off the device, either via the emergency button or the on/off switch. It comprises a parallel button that is triggered when the device is switched off and discharges the energy stored in the capacitors via a series of diodes and resistors through the system ground. This mechanism ensures the safe discharge of the stored energy and prevents the current from escaping toward the FES electrodes on the patient’s skin. In addition, ESP32 firmware was programmed to enforce a maximum-frequency lock for the boost converter. This precaution prevents the device from generating unwanted high voltages, which can potentially damage the user. Besides the mentioned mechanisms, a commercial power supply was used that safely regulates the 220 V AC mains voltage to 7 V DC. This ensures that electrical stimulation occurs within safe and controlled limits.

### Experiments

#### Patients

Two adult individuals with partial SCI who required a walking aid for locomotion were recruited. These participants were first informed about the aim of this study, read the informed consent form, and gave their verbal and signed consent at the Edmond and Lily Safra International Institute of Neurosciences (IIN-ELS) in Macaiba-RN, Brazil. This study was approved by the Research Ethics Committee from the Santos Dumont Institute (ISD) of Brazil (code number 53127921.2.0000.0129) and conducted following the Declaration of Helsinki.

The first participant (P1) was a 65-year-old woman with ASIA D SCI (C5) who fell off a ladder two years earlier. She walked with a conventional walker for short distances and used a wheelchair for medium and long distances. P1 performed only the cycling (pedaling) task.

The second participant (P2) was a 42-year-old man with ASIA D SCI (C4) who had suffered a car accident 1 year and 7 months earlier and relied on Canadian crutches for mobility. P2 performed only the walking task.

Both volunteers did not have increased muscle tone or comorbidities and were selected for participation in this study based on the following inclusion criteria: no cognitive, visual, or auditory impairment at baseline, and good communication skills. The exclusion criteria were the presence of joint injuries in the lower limbs, progressive diseases, skin injuries, fractures, osteoporosis, and pacemaker use.

#### Cycling task

**Fig. 3:**
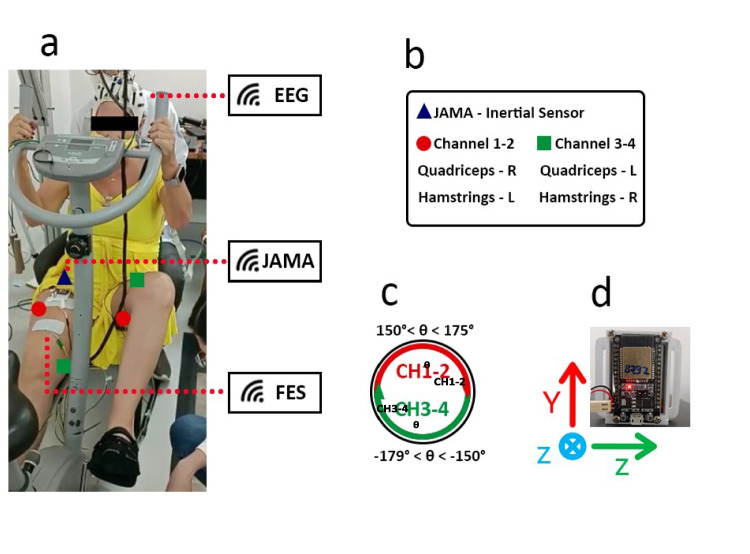
Setup used in the assisted cycling protocol (P1). **a** Participant’s posture, FES location, and devices; **b** Legend for electrodes and JAMA; **c** Identification range for pedaling phases and stimulated channels; **d** JAMA spatial orientation axes

P1 performed the cycling task, in which the algorithm differentiates the movement into two phases by using the IMU sensor as a reference (see Fig. 3(c)) to enable the electrical stimulation of the muscles at two points in time: 1) FES channels 1 and 2 for 150^*o*^ ≤ *θ* ≤ 175^*o*^; and 2) FES channels 3 and 4 for −179^*o*^ ≤ *θ* ≤ −150^*o*^. Consequently, the FES device alternately supports flexion and extension movements by stimulating the quadriceps in real-time.

The subject was instructed to sit on a stationary bicycle (Caloi^®^ S˜ao Paulo, Brazil) equipped with a non-motorized, unloaded pedal. The experiment was initiated according to the following steps: 1) preparation of the FES, EEG, and IMU; 2) signal acquisition at rest (baseline) for 5 min; 3) signal acquisition during voluntary pedaling at variable speeds for 5 min; and 4) signal acquisition during voluntary pedaling assisted by FES for 5 min.

During the preparation of the FES, the stimulation area was cleaned with alcohol and shaved. Electrodes (5 × 9 and 5 × 5, Carci^®^, S˜ao Paulo, Brazil) were then attached. The participants’ quadriceps and hamstring muscle groups were stimulated to induce and facilitate pedal movement [4]. To elicit robust contractions for functional movement, stimulation was applied prior to the protocol and gradually increased until discomfort occurred or until the therapist observed no further increase in muscle strength. During the protocol execution, the final stimulation intensity for each participant was set to 75% of the maximum value, with a frequency of 35 Hz and a pulse width of 300 *μ*s.

These muscle groups were chosen to facilitate the pedaling forward phase and pedaling backward phase, as in the studies by ([12, 40]). By applying this sequence alternately between the two lower limbs, the experimental subject produces the cycling movement.

The EEG cap was placed after cleaning the skin, and the conductive gel was used to achieve the impedance (10 kΩ). For determining the spatial references for lowerlimb positioning, the IMU sensor was positioned anteriorly and laterally under the fibular head of the right leg. Accelerometer and gyroscope data from this sensor were recorded at an acquisition rate of 20 Hz. Figure 3 shows a summary of the setup and pedaling cycle divided into two phases: −179^*o*^ to −150^*o*^ (right leg) and 150^*o*^ to 175^*o*^ (left leg). The sensor measured the current state (*θ*) while the participant performed the pedaling movement.

#### Walking task

During the walking task, P2 (4) underwent continuous phase detection and real-time activation of the electrical stimulation routine throughout the entire protocol (Fig. 5). The gait cycle was divided into four distinct phases: initial contact, mid-stance, terminal stance, and initial swing.

**Fig. 4:**
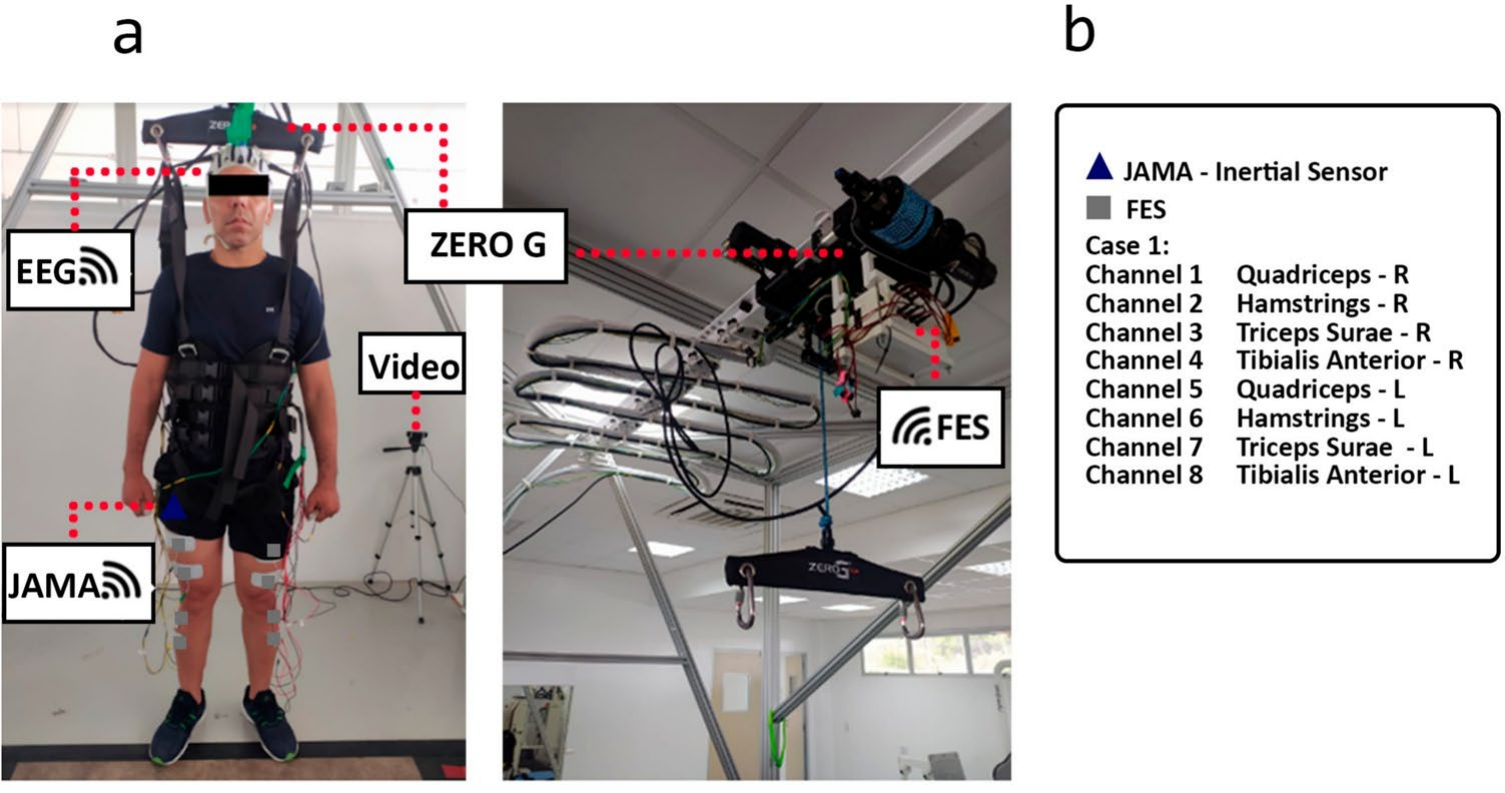
Setup used in the assisted gait protocol (P2). **a** Participant’s posture, FES location, and devices; **b** Legend for electrodes and JAMA

**Fig. 5:**
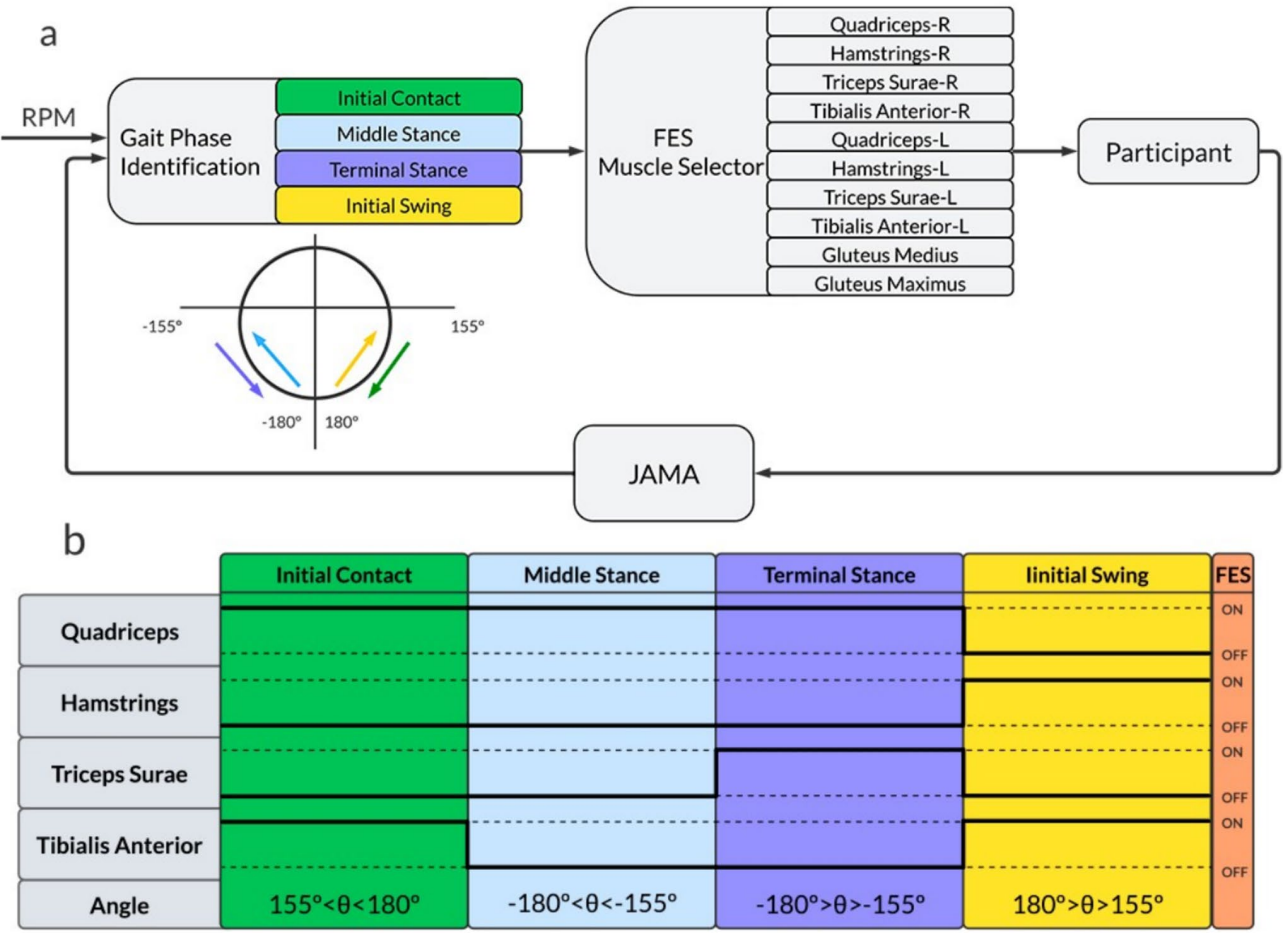
IMU sensor-based Human-Machine Interface for movement phase identification to provide a closed-loop between the user and the FES device. **a** block diagram, **b** activated muscles versus gait phases

The experimental setup for the gait activity is illustrated in Fig. 4. The muscle groups corresponding to each stimulation channel and the associated intensity levels were defined through the WEB interface, as illustrated in Fig. 5. In this configuration, the human–machine interface (HMI) operates in a closed-loop mode, where data from the IMU sensor are continuously processed to identify the current gait phase. Each detected phase then triggers the activation of a specific set of muscles, as shown in Fig. 5(b).

These muscles were chosen based on studies that observed the sequence of muscle activations in each subphase of gait through real-time surface electromyography [33]. Based on this, the electrostimulator was programmed to activate the channels corresponding to the muscles that should be active in each of these subphases: initial contact (quadriceps and tibialis anterior), mid-stance (quadriceps), terminal stance (quadriceps and triceps surae), and initial swing (hamstrings and tibialis anterior).

Overall, Fig. 5 illustrates the closed-loop operation of phase detection and electrical stimulation, showing the integration between sensor feedback, gait phase recognition, and muscle activation throughout the walking protocol.

The walking protocol was initiated using the following steps: 1) preparation of Zero G, FES, EEG, and IMU; 2) signal acquisition at rest (baseline) for 5 minutes; 3) signal acquisition during voluntary walking for 5 minutes; and 4) signal acquisition during FES-assisted walking for 5 minutes.

The gait-trained subjects wore a vest and partially supported their weight until they remained upright without bending their knees. The intervention was performed using the ZeroG^®^ system, which supported a certain percentage of body weight to ensure the participant’s safety during exercise. The support level was set at 10%.

EEG, FES, and IMU preparations were performed under the same conditions as the cycling protocol. The IMU sensor was set at 12 cm below the greater trochanter of the right femur. During the protocol, the quadriceps femoris, hamstrings, triceps surae, and tibialis anterior muscles were stimulated.

For this protocol, the walking movement was divided into four distinct phases: initial contact, middle stance, terminal stance, and initial swing. Through the WEB application, the therapist adjusted the angle range, stimulation intensity, and frequency, as well as the muscle groups to be recruited for each phase, based on the needs of individual participants.

### Evaluation

To ensure the reliability of EEG acquisition and minimize artifacts during FES-assisted tasks, active electrodes with integrated preamplification were used to reduce cable and environmental interference. The acquisition system operated on a battery-powered notebook, electrically isolated from the mains supply to avoid ground noise. Before recording, the scalp was cleaned with alcohol and conductive gel to maintain electrode–skin impedance below (10 kΩ), and the cap was carefully adjusted to prevent electrode movement during gait.

To understand the cortical effects of the proposed FES-based HMIs, relative EEG power changes during continuous pedaling or walking were quantified. EEG signals were pre-processed by applying a Common Average Reference (CAR) and a bandpass filter with a frequency range from 8 to 45 Hz to suppress unwanted noise and interference, preserving the brain rhythms of interest, as reported in [44]. The IMU signals were used to segment the EEG data into individual gait or pedaling cycles, where each cycle was considered a trial. Because of cycle duration varied across participants, each trial was resampled by interpolation to normalize its length to 0–100% of the cycle before averaging across trials. According to previous studies [34, 44], Cz provides the most discriminative information in terms of lower-limb movements. Hence, the power spectrum of the signal was calculated using the fast Fourier transform for each segment [44]. A Continuous Wavelet Transform (CWT) using the Morlet mother function was also calculated to obtain the joint time-frequency domain, and also the relative power changes during pedaling or walking with respect to the resting state [37]. Finally, topoplots corresponding to the average relative power changes for mu (8–13 Hz), low beta (13–20 Hz), and high beta (20–30 Hz) bands were obtained from all EEG locations for both walking and pedaling.

## Results and discussion

**Fig. 6:**
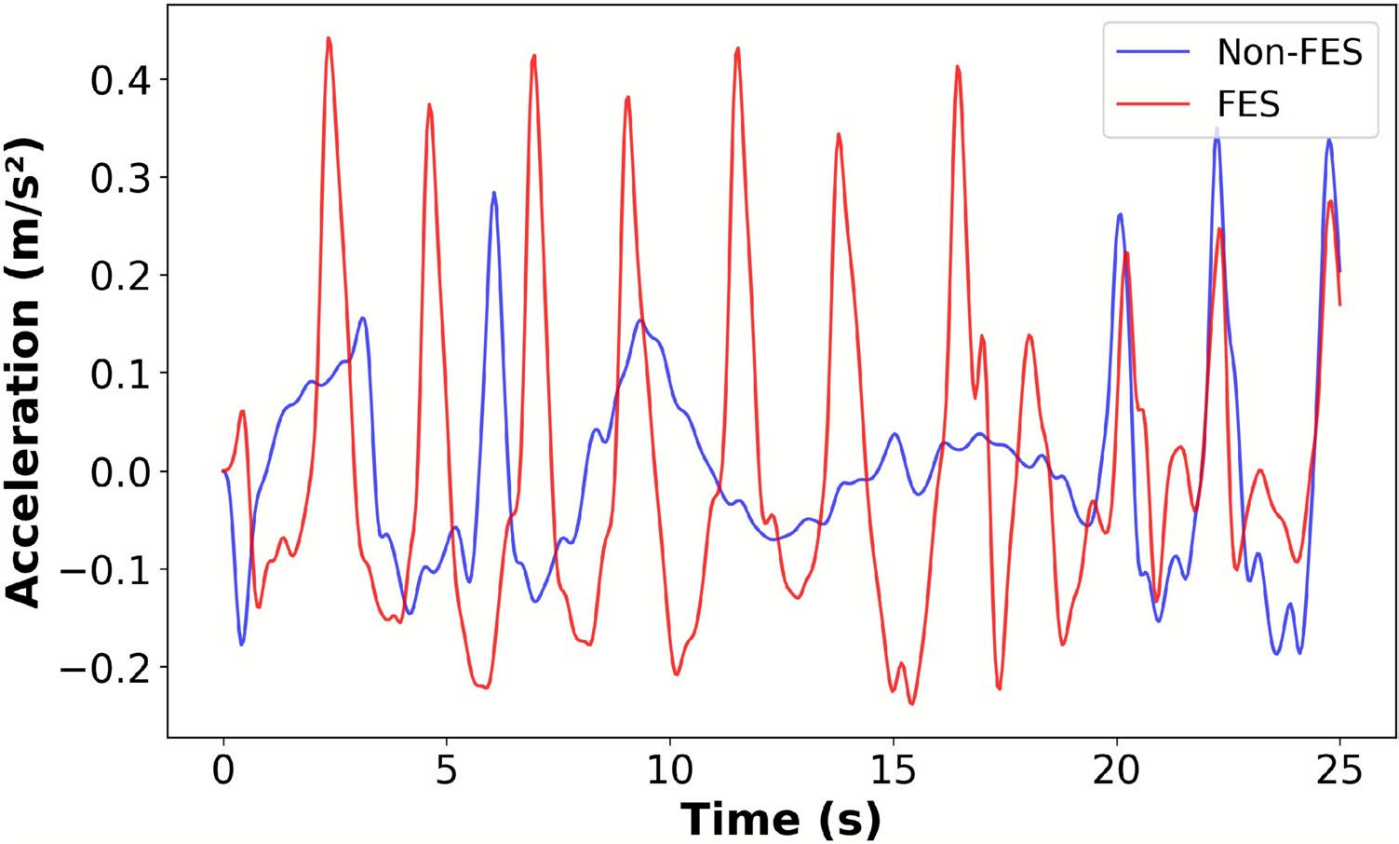
Inertial Motion Unit signals recorded while the SCI individual walked without and with the assistance of the proposed human-machine interface

The IMU sensor provided data demonstrating the impact of our proposed FES-based HMI on SCI individuals during walking and cycling. Improved walking was observed in SCI individuals, as shown in Fig. 6. The consistency of the movement cycles by using our HMI system compared with the non-assisted movement (Non-FES) suggests a more natural gait [18].

**Fig. 7:**
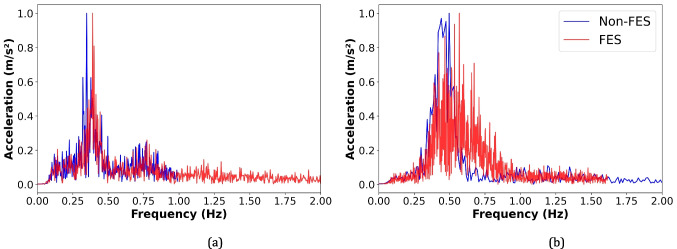
Movement frequency without and using the proposed IMU-based HMI and FES end-effector during (**a**) walking and (**b**) pedaling

**Fig. 8:**
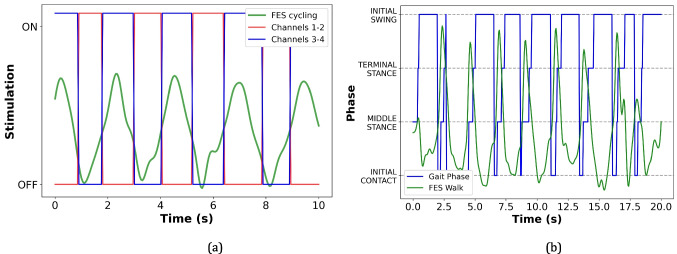
Channel activation during lower-limb tasks: **a** pedaling, where channels 1 and 2 correspond to the stimulation of the right quadriceps and left hamstring, and channels 3 and 4 correspond to the stimulation of the left quadriceps and right hamstring; **b** walking, showing the sequence of muscle group activation throughout the gait cycle

Figure 7a and b show that our FES-based HMI approach increased the movement frequency or speed, reaching approximately 0.4 Hz and 0.6 Hz for gait and pedaling cycles, respectively. This suggests that voluntary lower-limb movements of SCI individuals may be enhanced by using our system, increasing gait cycle repetitions and cadence. Figure 8a and b show a clear progression between phases during the FESbased HMI-assisted movement for cycling and walking. It is worth noting that our system automatically identifies with adequate accuracy the movement phase following the voluntary user’s motor intention in real-time in the associated angular range, facilitating FES stimulation and producing more natural transitions between phases.

Our results are supported by the literature, where different authors have used FES-based HMIs for gait rehabilitation in patients with neuromotor disabilities. For instance, da Stampacchia et al. reported that the use of this type of interface improves gait speed in post-SCI patients compared to conventional therapies [43]. On the other hand, Duffet et al. reported that the use of FES stimulators with lower limb tasks performed at home, such as cycling, accelerates the recovery process of lower limb mobility of post-SCI subjects, generating positive effects on gait [15]. In this context, our results suggest that assistance with FES strategically activated during gait cycles synchronously in an HMI may have these effects on gait rehabilitation, such as walking speed.

**Fig. 9:**
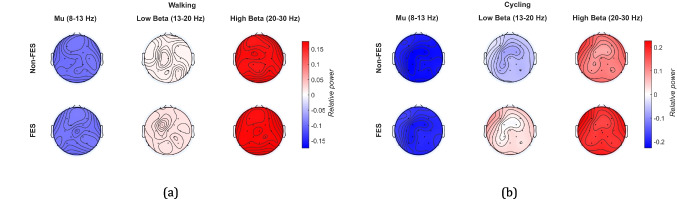
Topography distribution of the EEG relative power in FES and non-FES conditions during the execution of (**a**) walking and (**b**) cycling

Regarding neuronal behavior, Fig. 9 shows the topographic relative power changes with respect to the rest state of SCI individuals during walking and cycling tasks. Here, the topographies demonstrate a power decrease in the parieto-central cortex for the mu band, together with a power increase for the high beta band, which agrees with the results of Pfurtscheller and Neuper [34]. We did not find significant power differences between the assisted and non-assisted conditions, but power changes focused around Cz were observed, as previously reported in [34, 37].

Moreover, the frontal cortex over FC1 and FC3 specifically exhibited more concentrated power changes in the low beta band (see Fig. 9a). A similar finding was previously reported by Sinkjaer et al., who applied FES to feedback in healthy subjects [42]. Our findings may be associated with movement phase detection using the IMU signal in the proposed FES-based HMI, which, together with the ZeroG system, improved gait posture and performance. On the other hand, for the pedaling tasks, it was possible to demonstrate a greater power decrease for the mu band together with a power increase in the high beta band in the FES condition compared to the non-FES condition. This could be associated with an improvement in neuromuscular performance caused by FES, as similarly reported by Van der Scheer et al. [38].

**Fig. 10:**
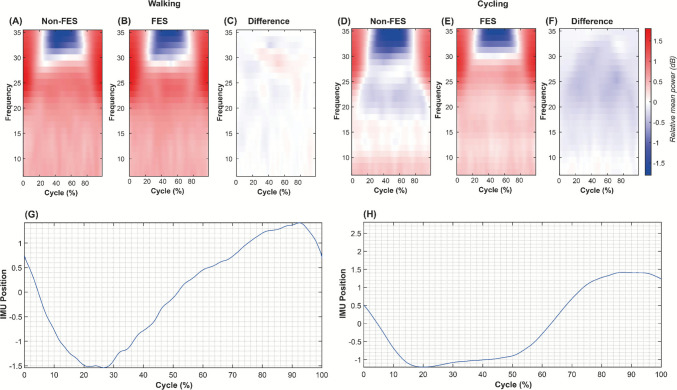
Grand average time-frequency representations of power changes in Cz electrode within the movement cycle relative to the movement cycle mean power (in dB) for (**A**) walking without FES assistance; (**B**) walking with FES assistance; (**C**) difference of walking with assistance and non-assistance with FES; (**D**) cycling without FES assistance; (**E**) cycling with FES assistance; (**F**) difference of cycling with assistance and non-assistance with FES. Each relative power was calculated considering the walking or pedaling cycles shown in (**G**) and (**H**), respectively

According to the literature, the brain regions around Cz provide the most relevant information associated with lower-limb movements [34, 37]. Figure 10 shows the power changes for the rest state in the joint time-frequency domain on cortical oscillations subserving cycling and walking. Our findings show power changes in the mu band during cycling movements, whereas power changes in the beta band between 30 and 35 Hz were observed in the middle of the gait cycle, which agrees with Storzer et al., who studied healthy subjects [44]. Comparing the assisted and non-assisted gait strategies, we observed power changes at approximately 30 Hz, as shown in Fig. 10(C), which decreased using our FES-based HMI. In contrast, during the FES-assisted cycling task, we observed a power decrease for the non-FES condition in the high beta (20–30 Hz), as shown in Fig. 10(F). Each relative power was calculated by considering each walk and pedal cycle during the protocol, as shown in Fig. 10(G) and (H), respectively.

**Fig. 11:**
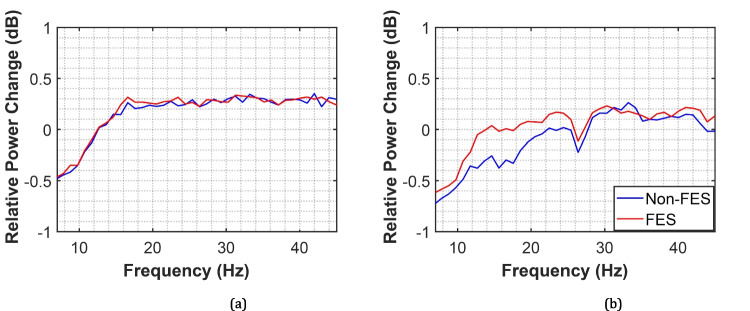
Grand average of the relative spectral power in dB to the rest period during continuous movement (**a**) walking; and (**b**) pedaling

To better understand cortical activity over Cz during walking and pedaling, Fig. 11 shows the power spectrum for each motor task. We observed a relative power decrease in the mu band during both FES- and non-FES-assisted movements, particularly during cycling (Fig. 11a and b). A relative power decrease was also found in the high-beta band during pedaling exercises, which agrees with the joint time–frequency analysis.

The findings of the current study were also observed in other related studies that showed the role of mu and beta rhythms during lower-limb movements [7, 19, 32, 37, 44]. Gait is a complex task that demands higher cognitive activity than cycling exercises. Note that similar power changes during both assisted and non-assisted gait were obtained in the joint time-frequency domain and frequency domain. However, we observed an evident difference in cycling exercises, mainly in the frequency range of 12–18 Hz, which may be associated with cognitive attention tasks [5, 27, 44]. It is worth mentioning that the SCI individual who performed cycling movements was seated in a comfortable chair; thus, the FES-assisted strategy facilitated her lower-limb movements, consequently demanding less attention.

Our results confirm that FES-based HMI systems can facilitate movement speed, but achieving a significant cortical impact is not possible. Therefore, researchers should explore brain signals from individuals with severe neural impairments and motor deficits to establish a direct pathway for triggering FES devices or other end effectors. The scientific community has shown interest in integrating EEG-based HMIs with IMUs, as it opens possibilities to develop alternative strategies for controlling end-devices, including FES, robotic exoskeletons, and serious games, among others. For instance, Hernandez-Rojas et al. [21] reported the feasibility of using FES-based BCIs for SCI people to command their hand opening and closing. Other approaches have related to FES for assistance of lower-limb. Le Guillou et al. realized a pedaling phase recognition system, which can be used to synchronously deliver FES to the legs [25]. However, the aforementioned studies did not employ HMI for (de) activate FES systems.

Coelho-Magalahes et al. designed a closed-loop control strategy to adjust FES amplitudes during pedaling [9]. Sijobert et al. employed a multichannel FES and sensors based on plantar pressure and IMUs to activate FES in one leg during gait in post-stroke patients [41]. Li et al. Implemented a neural network-based controller and sEMG to actuate foot dorsiflexion that was used by people with foot drop [26]. These approaches, although promising, did not incorporate FES for assisted assistance on both legs for each specific phase of movement. In this context, our system offers promising avenues for the development of therapeutic interventions with more natural movements using HMIs.

Our HMI relies on residual movements, which restrict its use to participants with partial SCI. One solution to overcome this limitation is to directly employ brain signals for lower-limb decoding and HMI control. Some strategies have explored this methodology, e.g. Luu et al. [29] proposed a system based on the kinematic decoding of gait through EEG signals, whereas Blanco-Diaz et al. [5] implemented the same strategy for reconstructing the pedaling movement. Moreover, deep learning-based techniques have been used for gait decoding, such as the studies proposed by Tortora et al. [46] with healthy subjects. On the other hand, other studies have demonstrated the promising use of BCIs when they are used in rehabilitation contexts with other lower limb assistive devices, such as exoskeletons [6].

This study presents different contributions in the fields of rehabilitation and neuroengineering, where it is important to highlight that the proposed solution, based on FES and IMU, demonstrated efficient functioning and flexibility, mainly when applied in cycling protocols. This could be because pedaling does not require dynamic or center-of-mass changes during execution which would allow easier decoding, thereby enhancing the impact between the FES and non-FES conditions [8]. The system effectively identified the movement phases using only one IMU, thereby simplifying the setup process. However, sensory feedback plays an important role in motor recovery and is often overlooked in the design of closed-loop systems for lower limb rehabilitation [6, 39]. For this reason, our system can generate sensory feedback in different muscles through multi-channel FES during each gait phase, and differences in cortical rhythms during pedaling and walking cycles were detected in the central cortex of the brain, showing feasibility for applications in BCIs.

This study presents some limitations due to the small sample size, thus, future research should focus on expanding the number and diversity of SCI individuals, including more severe motor impaired cases in a long-term training protocol. It will help understanding the generalization of our findings, and statistically confirms observed effects on functional and clinical outcomes longitudinally, and also in cortical modulation. It is important to determine whether the use of our system contributes to neuroplasticity, gait symmetry, muscle strength, and quality of life in individuals with spinal cord injury.

Brain related data in EEG signals will be better processed to reject artifacts and interferences that generally appear during walking, and can mask subtle cortical changes. In addition, the HMI relies on residual lower-limb movement to detect gait or pedaling phases based on inertial signals, which restricts its applicability to individuals without preserved voluntary mobility. Therefore, integrating brain signals-based gait or pedaling decoding may enable individuals without residual motor function to actively drive end-devices and practice physical exercises, exciting cortical motor areas. Other future work can explore adaptive control strategies using machine learning or reinforcement learning to modulate stimulation parameters, and enhance motor recruitment and increase user safety.

## Conclusion

The proposed FES-IMU-based HMI technology demonstrated promising results and good functioning in gait and cycling training protocols for volunteers with partial SCI. This proof of concept study revealed the technology’s flexibility in adapting to diverse movement tasks. The collected data suggest optimization of speed, cadence, and movement execution with FES assistance using only one IMU in the HMI composition. The multichannel FES hardware proved to be functional, enabling adaptability and automation in online and offline parameterization. Brain signal dynamics differed between FES and non-FES conditions during gait and pedaling, especially in the mu (8–13 Hz) and high-beta (20–30 Hz) bands, potentially correlated with movement correction caused by strategic FES activation. The results of this research open the door for further study of neural behaviors during lower limb tasks using FES and IMUs, aiming to restore mobility and rehabilitate people with neuromotor impairments, which greatly affect their daily activities such as walking or pedaling.
